# INS/CNS Deeply Integrated Navigation Method of Near Space Vehicles

**DOI:** 10.3390/s20205885

**Published:** 2020-10-17

**Authors:** Rongjun Mu, Hongchi Sun, Yuntian Li, Naigang Cui

**Affiliations:** School of Aerospace Science and Technology, Harbin Institute of Technology, Harbin 150001, China; murjun@163.com (R.M.); 15B918053@stu.hit.edu.cn (Y.L.); Cui_Naigang@163.com (N.C.)

**Keywords:** near space vehicles, integrated navigation, H-infinity filter, celestial navigation

## Abstract

Celestial navigation is required to improve the long-term accuracy preservation capability of near space vehicles. However, it takes a long time for traditional celestial navigation methods to identify the star map, which limits the improvement of the dynamic response ability. Meanwhile, the aero-optical effects caused by the near space environment can lead to the colorization of measurement noise, which affects the accuracy of the integrated navigation filter. In this paper, an INS/CNS deeply integrated navigation method, which includes a deeply integrated model and a second-order state augmented H-infinity filter, is proposed to solve these problems. The INS/CNS deeply integrated navigation model optimizes the attitude based on the gray image error function, which can estimate the attitude without star identification. The second-order state augmented H-infinity filter uses the state augmentation algorithm to whiten the measurement noise caused by the aero-optical effect, which can effectively improve the estimation accuracy of the H-infinity filter in the near space environment. Simulation results show that the proposed INS/CNS deeply integrated navigation method can reduce the computational cost by 50%, while the attitude accuracy is kept within 10” (3 σ). The attitude root mean square of the second-order state augmented H-infinity filter does not exceed 5”, even when the parameter error increases to 50%, in the near space environment. Therefore, the INS/CNS deeply integrated navigation method can effectively improve the rapid response ability of the navigation system and the filtering accuracy in the near space environment, providing a reference for the future design of near space vehicle navigation systems.

## 1. Introduction

Near space refers to the airspace 20 to 100 km above the surface [1]. Near space vehicles can cruise at a high speed in both the atmosphere and space. Compared with traditional aircraft, near space vehicles have been widely applied in space transportation, remote penetration, and so on, due to their advantages relating to launch costs and re-usability, among others [2,3]. Therefore, near space vehicles have drawn a great amount of attention, and many countries, such as the United States, Russia, China, France, and Germany, have conducted research on near space vehicles, such as the X-43A, X51A, X-37B, SHEFEX-1, SHEFEX-2, Avangard, and so on [4,5].

Near space vehicles fly so fast that attitude errors can greatly affect their position accuracy [6,7]. As the most accurate navigation method [8,9,10], celestial navigation is helpful for improving the long-term accuracy preservation capability of near space vehicles [11]. Celestial navigation calculates the attitude of aircraft by measuring stars which are firmly fixed in the inertial space, such that navigation error does not accumulate with time. Star sensors are widely used in modern celestial navigation systems, which capture images of stars and calculate the attitude according to the star point locations [12]. However, star sensors are vulnerable to environmental impacts. To improve the reliability of the navigation system, an inertial sensor is usually added to produce an integrated navigation system [13,14]. The INS/CNS integrated navigation system can be divided into two modes: Loose integration and tight integration [15]. In a loosely integrated navigation system, the inertial and star sensors work independently, where the measurement of the integrated navigation system is subject to the navigation error of both [16]. In a tightly integrated navigation system, the star sensor is no longer used for independent navigation calculation but, instead, is used as a sensor to measure the star vector; the system then sets up the measurement equation based on the star vector error [17,18]. The tightly integrated mode can save computational time at the sensor level, but it needs more time to update the measurements in the navigation filter. The computational efficiency of tightly integrated mode is close to loosely integrated mode in the whole navigation loop. Regardless of whether a loosely or tightly integrated navigation system is employed, star identification is necessary to identify navigation stars, which is the most time-consuming step [9,19]. The computational cost of the star sensor causes an attitude data delay [20]. When the vehicle speed is low and the attitude changes slowly, the attitude delay does not affect the navigation accuracy. However, the speed of near space vehicles is so high, and their mobility is so strong, that data delay has a significant impact on their navigation accuracy. Therefore, one solution is to simplify the celestial navigation algorithm and improve the computational efficiency of the star sensor [5,20].

Furthermore, the complex flow structures around the optical window of the star sensor produce target image offsets (called aero-optical effects), which is an important factor affecting the navigation accuracy of the near space vehicle [21,22]. The main problem of celestial navigation in the near space environment is the colored noise caused by aero-optical effects, which can cause the accuracy of the integrated navigation filter to decrease or even diverge [23,24].

At present, the Kalman filter is the most widely used technology in integrated navigation systems [25]. However, the Kalman filter makes some ideal assumptions in the filtering process, such as knowledge of the system model and system disturbances. The Kalman filtering algorithm assumes that the noise is white noise with zero mean and known covariance [26]; obviously, this assumption is no longer tenable in the near space environment. To deal with the uncertainty of noisy models, the H-infinity filter has been proposed. The H-infinity filter does not require the statistical characteristics of the noise model and has better robustness in the uncertainty system model [27]. Therefore, the H-infinity filter has great advantages over other filters in the near space environment.

Based on the above analyses, there are two problems that need to be solved: First, due to the strong mobility and high speed of near space vehicles, attitude data delay has a great impact on the navigation accuracy [1,5,20]. Therefore, the computational efficiency of the navigation algorithm needs to be improved, in order to adapt to the dynamic characteristics of near space vehicles. Second, the aero-optical effects affect the observation of the star sensor, leading to colorized measurement noise [21,23]. Therefore, the filter of the integrated navigation system needs to be improved using a colored noise model, in order to adapt to the near space environment.

To solve the above problems, an INS/CNS deeply integrated navigation model is proposed in this paper. In loosely and tightly integrated mode, IMU and star sensor work independently, but in deeply integrated mode, IMU will be involved in the calculation process of star sensor. IMU data is used to predict the navigation star and ensure that the algorithm can converge. In the deeply integrated navigation system, the star sensor only outputs a gray image (star identification is unnecessary). The attitude is obtained by optimizing the gray image error function, which is constructed with the help of IMU. Therefore, compared with the loosely and tightly integrated modes, the integration will be deeper in deeply integrated mode. Meanwhile, a state augmentation algorithm is introduced to whiten the measurement noise, which can effectively reduce the uncertainty of the measurement noise model. Then, a second-order state augmented H-infinity filter is proposed by using prior information of the colored noise, which can effectively improve the estimation accuracy of the near space vehicle navigation system.

This paper is organized as follows: In Section 2, the INS/CNS deeply integrated navigation model is proposed, to improve the computational efficiency of the star sensor. The second-order state augmented H-infinity filter is proposed in Section 3, to improve the navigation accuracy under colored noise conditions in the near space environment. Simulations are presented in Section 4, to demonstrate the performance of the proposed model. Finally, our conclusions are drawn in Section 5.

## 2. INS/CNS Deeply Integrated Model

The main idea of INS/CNS integrated navigation is to use the error between the measurement information of the star sensor and the prediction information of the INS to estimate the misalignment angle. The difference is the navigation information processing level; for example, a loosely integrated model deals with navigation information at the attitude angle level [28], while a tightly integrated model deals with navigation information at the star vector level [29]. Furthermore, the deeply integrated model deals with navigation information at the gray image level. The deeper the navigation information level is, the less processing links there are to the star sensor. Therefore, in the tightly integrated mode, the star sensor does not need to calculate the attitude. In the deeply integrated mode, star identification is only used to provide initial values at the beginning, the star sensor does not need to carry out star identification subsequently.

### 2.1. Gray Image Error Function

The gray image error function is the gray error between the measurement star image of the star sensor and the predicted star image by the inertial navigation system.

**Figure 1:**
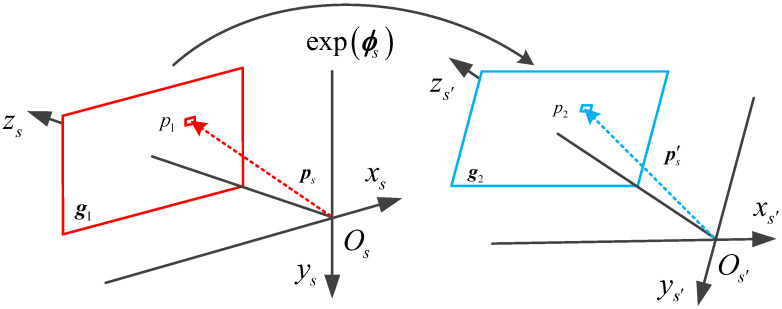
Construction process of the gray error function.

As shown in Figure 1, $g_{1}$ is the measurement star image of the star sensor; $g_{2}$ is the prediction star image of inertial navigation; $O_{s}x_{s}y_{s}z_{s}$ is the star sensor co-ordinate system; $O_{s^{\prime }}x_{s^{\prime }}y_{s^{\prime }}z_{s^{\prime }}$ is the virtual star sensor co-ordinate system based on INS prediction; $C_{s}^{s^{\prime }}$ is the transformation matrix between the star sensor and virtual star sensor co-ordinate systems; and $\phi_{s}$ is the misalignment angle of $C_{s}^{s^{\prime }}$, which satisfies $C_{s}^{s^{\prime }}=\exp\left(\phi_{s}\times \right)$. Suppose $p_{1}$ is a pixel in $g_{1}$ and $p_{s}$ is the projection vector of $p_{1}$ in the star sensor co-ordinate system, which satisfy [30]:

(1) $$p_{1}=\left[\begin{array}{c} u_{x}\\ u_{y}\\ 1 \end{array}\right]=\left[\begin{array}{ccc} f_{x} & 0 & c_{x}\\ 0 & f_{y} & c_{y}\\ 0 & 0 & 1 \end{array}\right]\left[\begin{array}{c} X/Z\\ Y/Z\\ 1 \end{array}\right]=A{\bar{p}}_{s},$$

where $u_{x}$ and $u_{y}$ represent the horizontal and vertical pixel co-ordinates, respectively; A is the internal parameter matrix of the star sensor; the parameters $f_{x}$, $f_{y}$, $c_{x}$, and $c_{y}$ are fixed after delivery; X, Y, and Z are the co-ordinates of $p_{s}$; and ${\bar{p}}_{s}$ is the normalized vector of $p_{s}$.

Due to the INS misalignment angle, $g_{2}$ is based on the virtual star sensor co-ordinate system, $p_{s}$ and ${p^{\prime }}_{s}$ are projection vectors of the same point in the star sensor and virtual star sensor co-ordinate system, and $p_{2}$ represents the pixel co-ordinates of ${p^{\prime }}_{s}$ in $g_{2}$, which satisfy

(2) $$p_{2}=A{\bar{p^{\prime }}}_{s}=AC_{s}^{s^{\prime }}{\bar{p}}_{s}=A\exp\left(\phi_{s}\times \right){\bar{p}}_{s}.$$

$I_{1}\left(p_{1}\right)$ is the gray value of point $p_{1}$ in $g_{1}$, $I_{2}\left(p_{2}\right)$ is the gray value of point $p_{2}$ in $g_{2}$, $p_{1}$ and $p_{2}$ can be regarded as projections of the same vector into different images, ${\hat{\phi }}_{s}$ is the estimate of $\phi_{s}$, the subscript *i* represents the number of pixels in the image, and $e_{i}\left({\hat{\phi }}_{s}\right)$ is the gray error function of a single point, which calculated as follows:

(3) $$e_{i}\left({\hat{\phi }}_{s}\right)=I_{1}\left(p_{1}\right)-I_{2}\left(p_{2}\right)=I_{1}\left(A{\bar{p}}_{s}\right)-I_{2}\left(A\exp\left({\hat{\phi }}_{s}\times \right){\bar{p}}_{s}\right).$$

According to the gray scale invariant, image transformation does not change the gray value of the same star point [31]. When ${\hat{\phi }}_{s}$ is close to the true value $\phi_{s}$, $e_{i}\left({\hat{\phi }}_{s}\right)$ will be close to zero. Considering all pixels in the image, the star sensor attitude estimation problem can be transformed into a non-linear optimization problem, as follows:

(4) $$\underset{{\hat{\phi }}_{s}}{\min}J\left({\hat{\phi }}_{s}\right)={\left\|e\left({\hat{\phi }}_{s}\right)\right\|}^{2},\;e\left({\hat{\phi }}_{s}\right)=\left[\begin{array}{c} e_{1}\left({\hat{\phi }}_{s}\right)\\ \ldots \\ e_{N}\left({\hat{\phi }}_{s}\right) \end{array}\right],$$

where N is the number of pixels to be optimized in the image and $e\left({\hat{\phi }}_{s}\right)$ is the gray image error function. The optimal estimate of $\phi_{s}$ is obtained when $J\left({\hat{\phi }}_{s}\right)$ reaches its minimum.

### 2.2. Attitude Optimization Algorithm Based on the Damped Newton Method

**Figure 2:**
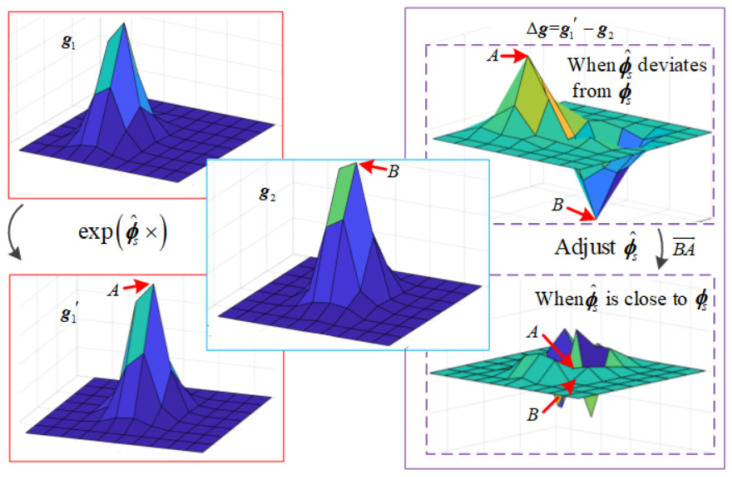
Fundamental principles of the deeply integrated model.

According to Equation (4), the principle of the deeply integrated model is to minimize the mean square error between two images by adjusting ${\hat{\phi }}_{s}$. To explain the principle of the deeply integrated model intuitively, suppose that there is only one star in the image. The physical meanings of $g_{1}$, $g_{2}$, and the gray image error function are shown in Figure 2.

At the beginning, star identification is needed to modify INS to make sure that ${\hat{\phi }}_{s}$ is small enough. Then, the star in $g_{1}$ and the star in $g_{2}$ will appear in the same star window as shown in Figure 2. There will be one obvious peak *A* and one obvious trough *B* in Δg above, adjust ${\hat{\phi }}_{s}$ in the direction of $\vec{BA}$. *A* and *B* will come closer and closer by iteration, and finally become the same as the Δg below.

According to the gray scale invariant,

(5) $$I_{1}\left(A{\bar{p}}_{s}\right)={I_{1}}^{\prime }\left(A\exp\left({\hat{\phi }}_{s}\times \right){\bar{p}}_{s}\right).$$

Equation (3) can be further written as

(6) $$e_{i}\left({\hat{\phi }}_{s}\right)=I_{1}\left(p_{1}\right)-I_{2}\left(p_{2}\right)={I_{1}}^{\prime }\left(A\exp\left({\hat{\phi }}_{s}\times \right){\bar{p}}_{s}\right)-I_{2}\left(A\exp\left({\hat{\phi }}_{s}\times \right){\bar{p}}_{s}\right)=\Delta I\left(p_{2}\right).$$

$\Delta I\left(p_{2}\right)$ is the gray value of point $p_{2}$ in Δg,the physical meaning of $e_{i}\left({\hat{\phi }}_{s}\right)$ can be understood as the value of the pixel co-ordinate $p_{2}$ in Δg. The purpose of global optimization is to minimize the mean square error of all points in Δg.

**Figure 3:**
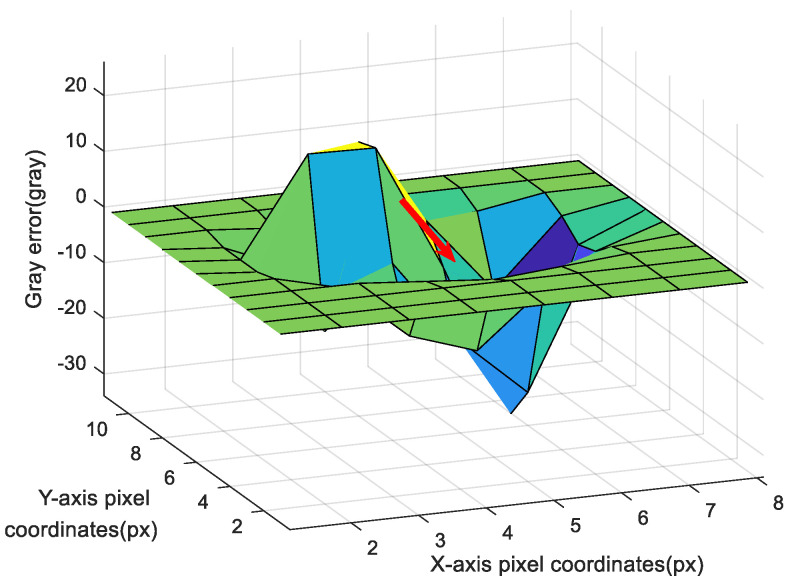
Optimization direction of gray error in a star window.

As shown in Figure 3, when ${\hat{\phi }}_{s}$ deviates from $\phi_{s}$, there will be an obvious peak and an obvious valley in Δg. The purpose of the deeply integrated model is to adjust the rotation relationship ${\hat{\phi }}_{s}$ between $g_{1}$ and $g_{2}$ until the peak and valley overlap, such that the gray error becomes close to zero. Obviously, the fastest adjustment direction is the red line in Figure 3, which is actually the gradient direction of $e_{i}\left({\hat{\phi }}_{s}\right)$. The gradient can be described as follows:

(7) $$\frac{\partial e_{i}\left({\hat{\phi }}_{s}\right)}{\partial {\hat{\phi }}_{s}}=\frac{\partial \left[I_{1}\left(p_{1}\right)-I_{2}\left(p_{2}\right)\right]}{\partial {\hat{\phi }}_{s}}=\frac{\partial \left[I_{1}\left(A{\bar{p}}_{s}\right)-I_{2}\left(A\exp\left({\hat{\phi }}_{s}\times \right){\bar{p}}_{s}\right)\right]}{\partial {\hat{\phi }}_{s}}=-\frac{\partial I_{2}\left(A\exp\left({\hat{\phi }}_{s}\times \right){\bar{p}}_{s}\right)}{\partial {\hat{\phi }}_{s}}.$$

It is difficult to calculate $\partial I_{2}/\partial {\hat{\phi }}_{s}$ directly, so an intermediate variable is introduced to decompose Equation (7). Suppose $\rho =\exp\left({\hat{\phi }}_{s}\times \right){\bar{p}}_{s}$ and ϑ=Aρ, then Equation (7) can be decomposed into:

(8) $$\frac{\partial e_{i}\left({\hat{\phi }}_{s}\right)}{\partial {\hat{\phi }}_{s}}=-\frac{\partial I_{2}}{\partial \vartheta }\frac{\partial \vartheta }{\partial \rho }\frac{\partial \rho }{\partial {\hat{\phi }}_{s}}.$$

Next, we calculate the three partial derivatives. $\partial I_{2}/\partial \vartheta$ is the pixel gradient of $g_{2}$ at ϑ. If one supposes that the pixel coordinate is $\vartheta ={\left[\begin{array}{cc} u_{a} & u_{b} \end{array}\right]}^{T}$, then the pixel gradient is

(9) $$\frac{\partial I_{2}}{\partial \vartheta }=\frac{1}{2}{\left[\begin{array}{c} I_{2}\left(u_{a}+1,u_{b}\right)-I_{2}\left(u_{a}-1,u_{b}\right)\\ I_{2}\left(u_{a},u_{b}+1\right)-I_{2}\left(u_{a},u_{b}-1\right) \end{array}\right]}^{T}.$$

Supposing that $\rho ={\left[\begin{array}{ccc} X_{\rho } & Y_{\rho } & Z_{\rho } \end{array}\right]}^{T}$, ∂ϑ/∂ρ can be calculated as follows:

(10) $$\frac{\partial \vartheta }{\partial \rho }=\left[\begin{array}{ccc} \frac{f_{x}}{Z_{\rho }} & 0 & -\frac{f_{x}X_{\rho }}{Z_{\rho }^{2}}\\ 0 & \frac{f_{y}}{Z_{\rho }} & -\frac{f_{y}Y_{\rho }}{Z_{\rho }^{2}} \end{array}\right].$$

It is difficult to calculate $\partial \rho /\partial {\hat{\phi }}_{s}$ directly, so the Baker–Campbell–Hausdorff formula is used to approximate $\partial \rho /\partial {\hat{\phi }}_{s}$, which satisfies [32]:

(11) $$\begin{array}{cc} \frac{\partial \rho }{\partial {\hat{\phi }}_{s}}= & \frac{\partial \exp\left({\hat{\phi }}_{s}\times \right){\bar{p}}_{s}}{\partial {\hat{\phi }}_{s}}=\underset{\delta {\hat{\phi }}_{s}\to 0}{\lim}\frac{\exp\left[\left({\hat{\phi }}_{s}+\delta {\hat{\phi }}_{s}\right)\times \right]{\bar{p}}_{s}-\exp\left({\hat{\phi }}_{s}\times \right){\bar{p}}_{s}}{\delta {\hat{\phi }}_{s}}\\ & =\underset{\delta {\hat{\phi }}_{s}\to 0}{\lim}\frac{\exp\left[\left(\kappa \delta {\hat{\phi }}_{s}\right)\times \right]\exp\left({\hat{\phi }}_{s}\times \right){\bar{p}}_{s}-\exp\left({\hat{\phi }}_{s}\times \right){\bar{p}}_{s}}{\delta {\hat{\phi }}_{s}}\approx \underset{\delta {\hat{\phi }}_{s}\to 0}{\lim}\frac{\left(\kappa \delta {\hat{\phi }}_{s}\right)\times \exp\left({\hat{\phi }}_{s}\times \right){\bar{p}}_{s}}{\delta {\hat{\phi }}_{s}}=-\rho \times \kappa \end{array}$$

According to the Baker–Campbell–Hausdorff formula [32],

(12) $$\left\{ \begin{array}{l} \kappa =\frac{\sin\chi }{\chi }I_{3\times 3}+\left(1-\frac{\sin\chi }{\chi }\right)\gamma \gamma^{T}+\frac{1-\cos\chi }{\chi }\left(\gamma \times \right)\\ \chi =\left|{\hat{\phi }}_{s}\right|,\gamma =\frac{{\hat{\phi }}_{s}}{\chi } \end{array}\right..$$

The Jacobian matrix,$J_{i}$, can be obtained by substituting Equations (9)–(11) into Equation (8), which satisfies:

(13) $$J_{i}=\frac{\partial e_{i}\left({\hat{\phi }}_{s}\right)}{\partial {\hat{\phi }}_{s}}=-\frac{\partial I_{2}}{\partial \vartheta }\frac{\partial \vartheta }{\partial \rho }\frac{\partial \rho }{\partial {\hat{\phi }}_{s}}=\frac{1}{2}{\left[\begin{array}{c} I_{2}\left(u_{a}+1,u_{b}\right)-I_{2}\left(u_{a}-1,u_{b}\right)\\ I_{2}\left(u_{a},u_{b}+1\right)-I_{2}\left(u_{a},u_{b}-1\right) \end{array}\right]}^{T}\left[\begin{array}{ccc} \frac{f_{x}}{Z_{\rho }} & 0 & -\frac{f_{x}X_{\rho }}{Z_{\rho }^{2}}\\ 0 & \frac{f_{y}}{Z_{\rho }} & -\frac{f_{y}Y_{\rho }}{Z_{\rho }^{2}} \end{array}\right]\left[\rho \times \kappa \right].$$

When there are *N* pixels to be optimized, each $J_{i}$ is stacked into a global Jacobian matrix $J\left({\hat{\phi }}_{s}\right)={\left[\begin{array}{ccc} J_{1}^{T} & \ldots & J_{N}^{T} \end{array}\right]}^{T}$, where $J_{i}$ is a 1×3 dimensional matrix and $J\left({\hat{\phi }}_{s}\right)$ is an N×3 dimensional matrix.

The damped Newton method is used to update ${\hat{\phi }}_{s}$, and $\Delta {\hat{\phi }}_{s}$ is calculated as follows:

(14) $$\left\{ \begin{array}{l} H_{J}\Delta {\hat{\phi }}_{s}=g_{J}\\ H_{J}=J^{T}\left({\hat{\phi }}_{s}\right)J\left({\hat{\phi }}_{s}\right)+\eta I\\ g_{J}=-J^{T}\left({\hat{\phi }}_{s}\right)e\left({\hat{\phi }}_{s}\right) \end{array}\right.,$$

where η is the damping coefficient, which can avoid singularities and make the iterative process more stable. Then, ${\hat{\phi }}_{s}={\hat{\phi }}_{s}+\Delta {\hat{\phi }}_{s}$ is used to modify ${\hat{\phi }}_{s}$ until it converges. It should be noted that the method does not always converge, when the initial value of ${\hat{\phi }}_{s}$ is larger than 2′, the method will diverge in simulation. Therefore, it is suggested that the initial value of ${\hat{\phi }}_{s}$ should be set within 2′ to ensure the convergence of the algorithm. Generally, the method only needs 3-4 iterations to achieve convergence.

Following which, $C_{n^{\prime }}^{n}$ can be calculated. $p_{s}$ and ${p^{\prime }}_{s}$ are the true and predicted values of the same vector, which satisfy

(15) $$\left\{ \begin{array}{l} p_{s}=C_{b}^{s}C_{n}^{b}p_{n}\\ {p^{\prime }}_{s}=C_{b}^{s}C_{n^{\prime }}^{b}p_{n}\\ {p^{\prime }}_{s}=C_{s}^{s^{\prime }}p_{s} \end{array}\right..$$

$C_{n^{\prime }}^{n}$ is calculated as follows:

(16) $$C_{n^{\prime }}^{n}={\left(C_{b}^{s}C_{n^{\prime }}^{b}\right)}^{-1}\exp\left({\hat{\phi }}_{s}\times \right)C_{b}^{s}C_{n^{\prime }}^{b}.$$

$C_{n^{\prime }}^{n}$ is used to modify $C_{n^{\prime }}^{b}$ to the CNS attitude result $C_{n}^{b}$, where $q_{cns}$ is the quaternion of $C_{n}^{b}$. As the CNS attitude, $q_{cns}$, has been obtained by the deeply integrated model, the next section will describe the filtering algorithm of the INS/CNS integrated navigation method.

## 3. Second-Order State Augmented H-Infinity Filter

There is a strong interaction between the aircraft and the surrounding airflow when the near space vehicle re-enters the atmosphere. This effect is called the aero-optical effect, which causes colorization of the attitude noise [23]. This section solves this problem by using a filtering approach.

### 3.1. Star Sensor Pixel Offset Model in the Near Space Environment

**Figure 4:**
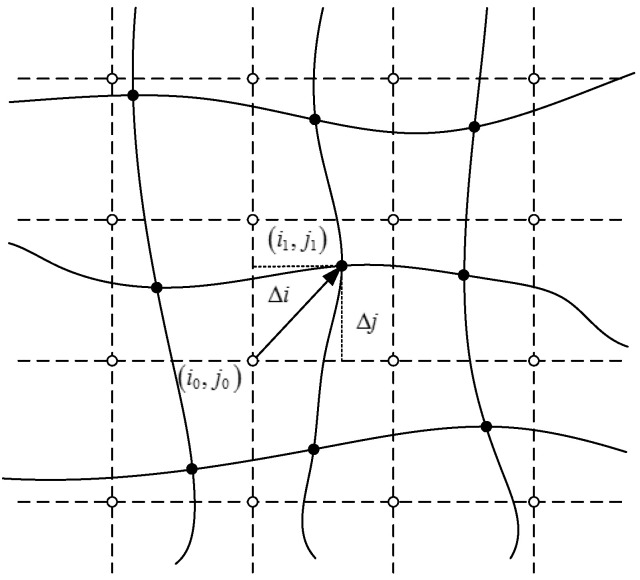
Sketch map of the pixel offset model.

When light travels through a rapidly varying flow field, the imaging position on the focal plane may be biased. The image distortion due to aero-optical effects can be described approximately as follows:

(17) $$I_{1}\left(i_{1},j_{1}\right)=I_{0}\left(i_{0}+\Delta i,j_{0}+\Delta j\right),$$

where $I_{0}\left(i_{0}+\Delta i,j_{0}+\Delta j\right)$ is the gray of reference image without aero-optical effects, $I_{1}\left(i_{1},j_{1}\right)$ is the gray of distorted image affected by aero-optical effects, $\left(i_{0},j_{0}\right)$ is the reference image co-ordinate, $\left(i_{1},j_{1}\right)$ is the distorted image co-ordinate, and Δi and Δj are the pixel offsets in the *X*-axis and *Y*-axis, respectively, caused by aero-optical effects. The pixel offset effect is shown in Figure 4, where the hollow point represents the original pixel position and the solid point represents the pixel position after offset.

It has been pointed out, in [24], that the pixel offset caused by aero-optical effects can be modeled approximately as follows:

(18) $$\left\{ \begin{array}{l} \Delta i\left(t\right)=a_{1}\sin\left(2\pi f_{1}t+\theta_{1}\right)\\ \Delta j\left(t\right)=a_{2}\sin\left(2\pi f_{2}t+\theta_{2}\right) \end{array}\right..$$

However, sinusoidal mathematical models are difficult to directly apply in the navigation systems of near space vehicles. This is because, in the actual flight environment, it is difficult to obtain the real-time phases $\theta_{1}$ and $\theta_{2}$. Therefore, in this paper, a recursive model is used to describe the pixel offset caused by aero-optical effects. The discrete sine sequence can be written as follows:

(19) sin(ωn)≈2cos(ω)sin(ω(n−1))−sin(ω(n−2)),

where ω is the digital angular frequency.

Therefore, the discrete recursive model of pixel offset can be obtained as follows:

(20) $$\left\{ \begin{array}{l} \Delta i\left(t_{k}\right)=2\cos\left(2\pi \frac{f_{1}}{fs}\right)\Delta i\left(t_{k-1}\right)-\Delta i\left(t_{k-2}\right)+\omega_{i}\left(t_{k}\right)\\ \Delta j\left(t_{k}\right)=2\cos\left(2\pi \frac{f_{2}}{fs}\right)\Delta j\left(t_{k-1}\right)-\Delta j\left(t_{k-2}\right)+\omega_{j}\left(t_{k}\right) \end{array}\right.,$$

where fs is the sampling frequency, and $\omega_{i}\left(t_{k}\right)$ and $\omega_{j}\left(t_{k}\right)$ are zero-mean white noise sequences. This modeling method only needs to know the frequency, which improves the feasibility of engineering applications.

### 3.2. Second-Order State Augmented H-Infinity Filtering Model

The H-infinity filter can obtain the optimal estimation of state variables under the condition of noise with unknown statistics [33,34]. According to the characteristics of the near space environment, the H-infinity filter can be improved based on the prior information of $V_{k}$. Specific improvements are presented in the following sections.

#### 3.2.1. Measurement Noise Whitening

Suppose the filter model is

(21) $$\left\{ \begin{array}{l} X_{k}=\Phi_{k/k-1}X_{k-1}+\Gamma_{k-1}W_{k-1}\\ Z_{k}=H_{k}X_{k}+V_{k} \end{array}\right.,$$

where $V_{k}$ is the observation noise of the star sensor in the near space environment, which includes the two parts $V_{k}=V_{c,k}+V_{w,k}$, in which $V_{c,k}$ is the colored noise part and $V_{w,k}$ is the white noise part ($E\left[V_{w,k}\right]=0$, $E\left[V_{w,k}V_{w,j}^{T}\right]=R_{w,k}\delta_{kj}$). The mechanisms of $V_{c,k}$ and $V_{w,k}$ are different, so they are not related. $V_{c,k}$ can be approximated as a second-order Markov process, which can be described as $V_{c,k}=\alpha_{1}V_{c,k-1}+\alpha_{2}V_{c,k-2}+\xi_{k}$, where $\xi_{k}$ is the driving white noise ($E\left[\xi_{k}\right]=0,E\left[\xi_{k}\xi_{j}^{T}\right]=R_{c,k}\delta_{kj}$). X and Φ can be described as follows:

(22) $$X={\left[\begin{array}{ccccc} \phi^{T} & {\left(\delta v_{n}\right)}^{T} & {\left(\delta p\right)}^{T} & {\left(\varepsilon_{b}\right)}^{T} & {\left(\nabla_{b}\right)}^{T} \end{array}\right]}^{T},\Phi \approx I+Ft.$$

where

(23) $$F=\left[\begin{array}{ccccc} -\left[\omega_{in}^{n}\times \right] & M_{v} & M_{1}+M_{2} & -C_{b}^{n} & 0_{3\times 3}\\ \left[f_{ib}^{n}\times \right] & -\left[\left(2\omega_{ie}^{n}+\omega_{en}^{n}\right)\times \right]+\left[v\times \right]M_{v} & M_{g}+\left[v\times \right]\left(2M_{1}+M_{2}\right) & 0_{3\times 3} & C_{b}^{n}\\ 0_{3\times 3} & M_{pv} & M_{pp} & 0_{3\times 3} & 0_{3\times 3}\\ 0_{3\times 3} & 0_{3\times 3} & 0_{3\times 3} & 0_{3\times 3} & 0_{3\times 3}\\ 0_{3\times 3} & 0_{3\times 3} & 0_{3\times 3} & 0_{3\times 3} & 0_{3\times 3} \end{array}\right].$$

(24) $$M_{1}=\left[\begin{array}{ccc} 0 & 0 & 0\\ -\omega_{ie}\sin L & 0 & 0\\ \omega_{ie}\cos L & 0 & 0 \end{array}\right],M_{2}=\left[\begin{array}{ccc} 0 & 0 & \frac{v_{N}}{{\left(R_{M}+h\right)}^{2}}\\ 0 & 0 & \frac{-v_{E}}{{\left(R_{N}+h\right)}^{2}}\\ \frac{v_{E}\sec^{2}L}{\left(R_{N}+h\right)} & 0 & \frac{-v_{E}\tan L}{{\left(R_{N}+h\right)}^{2}} \end{array}\right],M_{v}=\left[\begin{array}{ccc} 0 & -\frac{1}{R_{M}+h} & 0\\ \frac{1}{R_{N}+h} & 0 & 0\\ \frac{\tan L}{R_{N}+h} & 0 & 0 \end{array}\right].$$

(25) $$M_{pv}=\left[\begin{array}{ccc} 0 & \frac{1}{R_{M}+h} & 0\\ \frac{\sec L}{R_{N}+h} & 0 & 0\\ 0 & 0 & 1 \end{array}\right],M_{pp}=\left[\begin{array}{ccc} 0 & 0 & \frac{-v_{N}}{{\left(R_{M}+h\right)}^{2}}\\ \frac{v_{E}\sec L\tan L}{R_{N}+h} & 0 & \frac{-v_{E}\sec L}{{\left(R_{N}+h\right)}^{2}}\\ 0 & 0 & 0 \end{array}\right].$$

The first- and second-order terms of the noise sequence $V_{c,k}$ can be expressed as follows:

(26) $$\left[\begin{array}{c} V_{c,k}\\ V_{c,k-1} \end{array}\right]=\left[\begin{array}{cc} \alpha_{1}I & \alpha_{2}I\\ I & 0 \end{array}\right]\left[\begin{array}{c} V_{c,k-1}\\ V_{c,k-2} \end{array}\right]+\left[\begin{array}{c} \xi_{k}\\ 0 \end{array}\right].$$

$V_{c,k-1}$ and $V_{c,k-2}$ can be extended into the state parameters to form the second-order augmented state parameters and, so the measurement equation can be expressed as follows:

(27) $$Z_{k}=H_{k}X_{k}+V_{c,k}+\alpha_{1}V_{c,k-1}+\alpha_{2}V_{c,k-2}+\xi_{k}=\left[\begin{array}{ccc} H_{k} & \alpha_{1}I & \alpha_{2}I \end{array}\right]\left[\begin{array}{c} X_{k}\\ V_{c,k-1}\\ V_{c,k-2} \end{array}\right]+\left(V_{w,k}+\xi_{k}\right).$$

The state equation can be expressed as follows:

(28) $$\left[\begin{array}{c} X_{k}\\ V_{c,k-1}\\ V_{c,k-2} \end{array}\right]=\left[\begin{array}{ccc} \Phi_{k/k-1} & 0 & 0\\ 0 & \alpha_{1}I & \alpha_{2}I\\ 0 & I & 0 \end{array}\right]\left[\begin{array}{c} X_{k-1}\\ V_{c,k-2}\\ V_{c,k-3} \end{array}\right]+\left[\begin{array}{ccc} \Gamma_{k-1} & 0 & 0\\ 0 & I & 0\\ 0 & 0 & I \end{array}\right]\left[\begin{array}{c} W_{k-1}\\ \xi_{k}\\ 0 \end{array}\right].$$

Suppose

(29) $$\left\{ \begin{array}{l} X_{k}^{a}=\left[\begin{array}{c} X_{k}\\ V_{c,k-1}\\ V_{c,k-2} \end{array}\right],\Phi_{k/k-1}^{a}=\left[\begin{array}{ccc} \Phi_{k/k-1} & 0 & 0\\ 0 & \alpha_{1}I & \alpha_{2}I\\ 0 & I & 0 \end{array}\right],\Gamma_{k-1}^{a}=\left[\begin{array}{ccc} \Gamma_{k-1} & 0 & 0\\ 0 & I & 0\\ 0 & 0 & I \end{array}\right]\\ W_{k-1}^{a}=\left[\begin{array}{c} W_{k-1}\\ \xi_{k}\\ 0 \end{array}\right],H_{k}^{a}=\left[\begin{array}{ccc} H_{k} & \alpha_{1}I & \alpha_{2}I \end{array}\right],V_{k}^{a}=V_{w,k}+\xi_{k} \end{array}\right..$$

Then, the second-order state augmented model (after whitening) can be written as follows:

(30) $$\left\{ \begin{array}{l} X_{k}^{a}=\Phi_{k/k-1}^{a}X_{k-1}^{a}+\Gamma_{k-1}^{a}W_{k-1}^{a}\\ Z_{k}=H_{k}^{a}X_{k}^{a}+V_{k}^{a} \end{array}\right..$$

The noise characteristics of $W_{k}^{a}$ and $V_{k}^{a}$ can be analyzed, which satisfy

(31) $$\left\{ \begin{array}{l} E\left[W_{k}^{a}\right]=0,E\left[V_{k}^{a}\right]=0\\ E\left[W_{k}^{a}{\left(W_{j}^{a}\right)}^{T}\right]=E\left[\left[\begin{array}{c} W_{k-1}\\ \xi_{k}\\ 0 \end{array}\right]\left[\begin{array}{ccc} W_{k-1}^{T} & \xi_{k}^{T} & 0 \end{array}\right]\right]=\left[\begin{array}{ccc} Q_{k} & 0 & 0\\ 0 & R_{c,k} & 0\\ 0 & 0 & 0 \end{array}\right]\delta_{kj}\\ E\left[V_{k}^{a}{\left(V_{j}^{a}\right)}^{T}\right]=E\left[V_{w,k}V_{w,j}^{T}+\xi_{k}\xi_{j}^{T}\right]=\left(R_{w,k}+R_{c,k}\right)\delta_{kj}\\ E\left[W_{k}^{a}{\left(V_{j}^{a}\right)}^{T}\right]=E\left[\left[\begin{array}{c} W_{k-1}\\ \xi_{k}\\ 0 \end{array}\right]\left(V_{w,j}^{T}+\xi_{j}^{T}\right)\right]=\left[\begin{array}{c} 0\\ R_{c,k}\\ 0 \end{array}\right]\delta_{kj} \end{array}\right..$$

Although $V_{k}^{a}$ has been converted to white noise, the cost is that the system noise $W_{k}^{a}$ and measured noise $V_{k}^{a}$ become dependent ($E\left[W_{k}^{a}{\left(V_{j}^{a}\right)}^{T}\right]\neq 0$). This is because the prior model of the colored noise is added to the state quantity and the colored noise $V_{k}^{a}$ is affected by the driving white noise $\xi_{k}$, which leads to correlation between $W_{k}^{a}$ and $V_{k}^{a}$.

#### 3.2.2. Decorrelation of System Noise and Measurement Noise in the Second-Order Augmented Model

Equation (31) can be abbreviated as follows:

(32) $$\left\{ \begin{array}{l} E\left[W_{k}^{a}{\left(W_{j}^{a}\right)}^{T}\right]=Q_{k}^{a}\delta_{kj}\\ E\left[V_{k}^{a}{\left(V_{j}^{a}\right)}^{T}\right]=R_{k}^{a}\delta_{kj}\\ E\left[W_{k}^{a}{\left(V_{j}^{a}\right)}^{T}\right]=S_{k}\delta_{kj} \end{array}\right..$$

According to Equation (30), $0=J_{k-1}\left(Z_{k-1}-H_{k-1}^{a}X_{k-1}^{a}+V_{k-1}^{a}\right)$, where $J_{k-1}$ is an arbitrary matrix. The state equation can be organized as

(33) $$\begin{array}{cc} X_{k}^{a} & =\Phi_{k/k-1}^{a}X_{k-1}^{a}+\Gamma_{k-1}^{a}W_{k-1}^{a}+J_{k-1}\left(Z_{k-1}-H_{k-1}^{a}X_{k-1}^{a}+V_{k-1}^{a}\right)\\ & =\left(\Phi_{k/k-1}^{a}-J_{k-1}H_{k-1}^{a}\right)X_{k-1}^{a}+J_{k-1}Z_{k-1}+\left(\Gamma_{k-1}^{a}W_{k-1}^{a}-J_{k-1}V_{k-1}^{a}\right)=\Phi_{k/k-1}^{*}X_{k-1}^{a}+J_{k-1}Z_{k-1}+W_{k-1}^{*} \end{array}$$

where

(34) $$\left\{ \begin{array}{l} \Phi_{k/k-1}^{*}=\Phi_{k/k-1}^{a}-J_{k-1}H_{k-1}^{a}\\ W_{k-1}^{*}=\Gamma_{k-1}^{a}W_{k-1}^{a}-J_{k-1}V_{k-1}^{a} \end{array}\right..$$

The covariance matrix of system noise and measurement noise is as follows:

(35) $$E\left[W_{k}^{*}{\left(V_{j}^{a}\right)}^{T}\right]=E\left[\Gamma_{k}^{a}W_{k}^{a}{\left(V_{j}^{a}\right)}^{T}-J_{k}V_{k}^{a}{\left(V_{j}^{a}\right)}^{T}\right]=\left(\Gamma_{k}^{a}S_{k}-J_{k}R_{k}^{a}\right)\delta_{kj}.$$

Obviously, if $J_{k}$ satisfies $\Gamma_{k}^{a}S_{k}-J_{k}R_{k}^{a}=0$, $W_{k}^{*}$ and $V_{k}^{a}$ are no longer relevant and, so, $J_{k}$ should satisfy

(36) $$J_{k}=\Gamma_{k}^{a}S_{k}{\left(R_{k}^{a}\right)}^{-1}.$$

The variance matrix of $W_{k}^{*}$ is

(37) $$\begin{array}{l} E\left[W_{k}^{*}{\left(W_{j}^{*}\right)}^{T}\right]=\left(\Gamma_{k}^{a}Q_{k}^{a}{\left(\Gamma_{k}^{a}\right)}^{T}+J_{k}R_{k}^{a}J_{k}^{T}-\Gamma_{k}^{a}S_{k}J_{k}^{T}-J_{k}S_{k}^{T}{\left(\Gamma_{k}^{a}\right)}^{T}\right)\delta_{kj}\\ =\left(\Gamma_{k}^{a}Q_{k}^{a}{\left(\Gamma_{k}^{a}\right)}^{T}-J_{k}S_{k}^{T}{\left(\Gamma_{k}^{a}\right)}^{T}\right)\delta_{kj}=\left(\Gamma_{k}^{a}Q_{k}^{a}{\left(\Gamma_{k}^{a}\right)}^{T}-J_{k}R_{k}^{a}J_{k}^{T}\right)\delta_{kj} \end{array}$$

If $Q_{k}^{*}=\Gamma_{k}^{a}Q_{k}^{a}{\left(\Gamma_{k}^{a}\right)}^{T}-J_{k}R_{k}^{a}J_{k}^{T}$, the second-order state augmented model (after decorrelation) can be expressed as follows:

(38) $$\left\{ \begin{array}{l} X_{k}^{a}=\Phi_{k/k-1}^{*}X_{k-1}^{a}+J_{k-1}Z_{k-1}+W_{k-1}^{*}\\ Z_{k}=H_{k}^{a}X_{k}^{a}+V_{k}^{a} \end{array}\right.,$$

where

(39) $$\left\{ \begin{array}{l} E\left[W_{k}^{*}\right]=0,E\left[V_{k}^{a}\right]=0,E\left[W_{k}^{*}{\left(V_{j}^{a}\right)}^{T}\right]=0\\ E\left[W_{k}^{*}{\left(W_{j}^{*}\right)}^{T}\right]=Q_{k}^{*}\delta_{kj},E\left[V_{k}^{a}{\left(V_{j}^{a}\right)}^{T}\right]=R_{k}^{a}\delta_{kj} \end{array}\right..$$

Suppose the estimator of the H-infinity Filter, after expanding its dimension, is

(40) $$y_{k}={Τ}_{k}X_{k}=\left[\begin{array}{cc} {Τ}_{k} & 0 \end{array}\right]\left[\begin{array}{c} X_{k}\\ V_{c,k-1}\\ V_{c,k-2} \end{array}\right]={Τ}_{k}^{a}X_{k}^{a}.$$

A second-order state augmented H-infinity filter can be obtained by substituting Equation (38) and Equation (40) into the standard H-infinity formula, thus satisfying

(41) $$\left\{ \begin{array}{l} {\tilde{\Omega }}_{k}^{a}={\left({Τ}_{k}^{a}\right)}^{T}\Omega_{k}{Τ}_{k}^{a}\\ X_{k}^{a}=\Phi_{k/k-1}^{*}X_{k-1}^{a}+J_{k-1}Z_{k-1}+\Phi_{k/k-1}^{*}K_{k-1}\left(Z_{k-1}-H_{k-1}^{a}X_{k-1}^{a}\right)\\ K_{k-1}=P_{k-1}^{a}{\left[I-\theta {\tilde{\Omega }}_{k}P_{k-1}^{a}+{\left(H_{k-1}^{a}\right)}^{T}{\left(R_{k-1}^{a}\right)}^{-1}H_{k-1}^{a}P_{k-1}^{a}\right]}^{-1}H_{k-1}^{a}{\left(R_{k-1}^{a}\right)}^{-1}\\ P_{k}^{a}=\Phi_{k/k-1}^{*}P_{k-1}^{a}{\left[I-\theta {\tilde{\Omega }}_{k}P_{k-1}^{a}+{\left(H_{k-1}^{a}\right)}^{T}{\left(R_{k-1}^{a}\right)}^{-1}H_{k-1}^{a}P_{k-1}^{a}\right]}^{-1}{\left(\Phi_{k/k-1}^{*}\right)}^{T}+Q_{k-1}^{*} \end{array}\right..$$

## 4. Results and Discussion

### 4.1. Simulation Verification of the INS/CNS Deeply Integrated Model

#### 4.1.1. Algorithm Robustness Verification

The optimization object of the deeply integrated model is the global gray error of all navigation stars, where the optimization result is the optimal solution (in the least-squares sense). Theoretically, the mismatching of individual stars does not affect the final result; this was verified by simulation.

**Figure 5:**
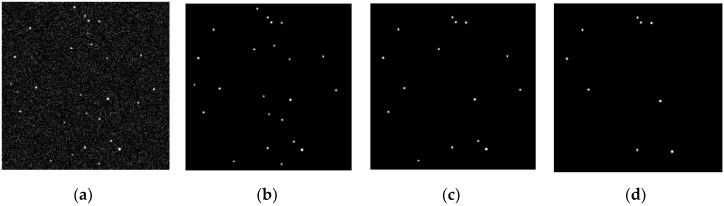
Initial star map for each working condition: (**a**) Star map observation by the star sensor; (**b**) prediction star map obtained under low uncertainty conditions; (**c**) prediction star map obtained under medium uncertainty conditions; and (**d**) prediction star map obtained under high uncertainty conditions (all star maps had enhanced contrast for ease of viewing).

The Smithsonian Astrophysical Observatory (SAO) catalog was used for simulation validation, where precession and nutation were compensated for by the IAU1980 model, aberration only considered first-order corrections, and polar shift correction was provided by the International Earth Rotation Service (IERS). As shown in Figure 5, the simulation was validated under three working conditions: Low, medium, and high uncertainty.

**Figure 6:**
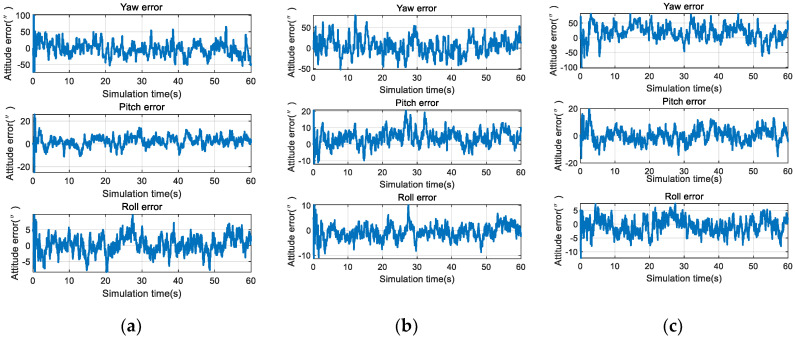
Attitude error curve for each working condition: (**a**) Attitude error curve under low uncertainty conditions; (**b**) attitude error curve under medium uncertainty conditions; and (**c**) attitude error curve under high uncertainty conditions.

The attitude calculation results obtained under the three conditions are shown in Figure 6.

From Figure 6, it can be seen that, even under high uncertainty (where the INS only correctly predicted 30% of the navigation stars), the deeply integrated algorithm still converged, and the accuracies under the three conditions were only slightly different.

To explain this phenomenon, suppose $e_{a}\left({\hat{\phi }}_{s}\right)$ ($1\leq a\leq N_{s}$) represents the gray error function of correctly matched pixels and $e_{b}\left({\hat{\phi }}_{s}\right)$ ($N_{s}+1\leq b\leq N$) represents the gray error function of incorrectly matched pixels. After the star point $p_{1}$ in $g_{1}$ is transformed into ${g_{1}}^{\prime }$ by $\exp\left({\hat{\phi }}_{s}\times \right)$, there will be no star point at the corresponding position $p_{2}$ in $g_{2}$ if the star points between $g_{1}$ and $g_{2}$ do not match. $e_{b}\left({\hat{\phi }}_{s}\right)$ will be equal to $I_{1}\left(p_{1}\right)$, which can be recorded as $C_{b}$. Obviously, $C_{b}$ is a constant that does not change with ${\hat{\phi }}_{s}$. The optimal objective is equivalent to

(42) $$\underset{{\hat{\phi }}_{s}}{\min}J\left({\hat{\phi }}_{s}\right)={\left\|e\left({\hat{\phi }}_{s}\right)\right\|}^{2}=\sum_{a=1}^{N_{s}}e_{a}^{2}\left({\hat{\phi }}_{s}\right)+\sum_{b=N_{s}+1}^{N}e_{b}^{2}\left({\hat{\phi }}_{s}\right)=\sum_{i=1}^{N_{s}}e_{a}^{2}\left({\hat{\phi }}_{s}\right)+\sum_{j=N_{s}+1}^{N}C_{b}^{2}\Leftrightarrow \underset{{\hat{\phi }}_{s}}{\min}J\left({\hat{\phi }}_{s}\right)=\sum_{i=1}^{N_{s}}e_{a}^{2}\left({\hat{\phi }}_{s}\right).$$

Equation (37) shows that the constant term does not affect the final optimization result, such that optimizing all stars is equivalent to only optimizing the correctly matched stars, which means that unmatched stars do not affect the optimization result.

#### 4.1.2. Comparison of Different Integrated Models

**Figure 7:**
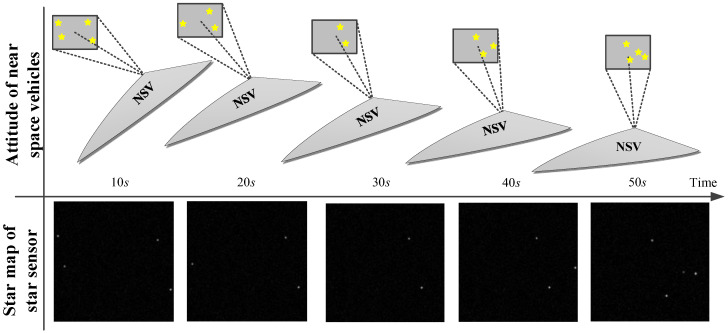
Simulation design.

Star identification is not necessary in the deeply integrated mode and the navigation system can still work when the number of navigation stars is less than three. Therefore, the simulation should cover three conditions: The number of navigation satellites is more than three, equal to three, and less than three. The star map of the star sensor in the simulation is shown in Figure 7.

**Figure 8:**
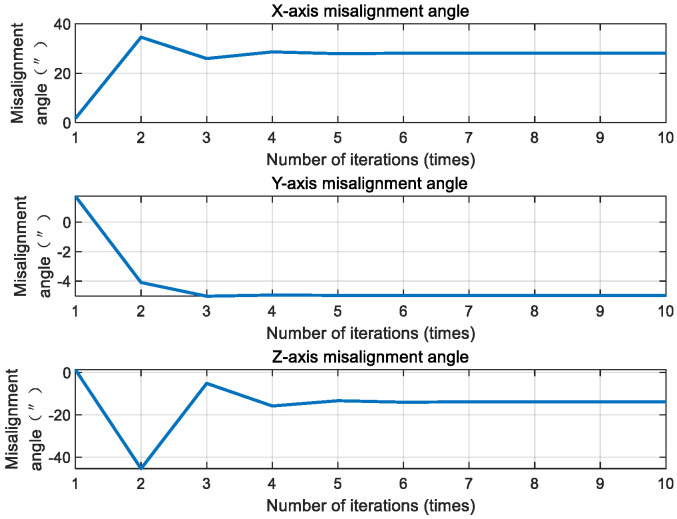
Iterative optimization results of the deeply integrated mode.

The damped Newton method was used to optimize the misalignment angle iteratively. Taking the time t=10 s as an example, the iterative optimization results are shown in Figure 8.

**Figure 9:**
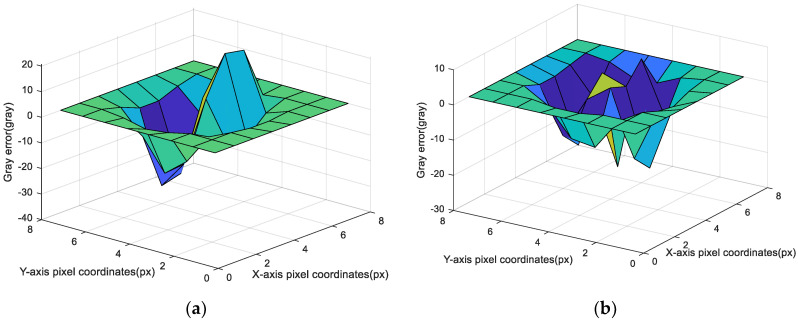
Iterative results of image gray error in a star window: (**a**) Image gray error of the first iteration; and (**b**) image gray error of the fourth iteration.

As shown in Figure 8, the attitude could reach convergence after only 3–4 iterations. Compared with the first and fourth iteration optimization results, the gray image error is shown in Figure 9.

As shown in Figure 9, ${\hat{\phi }}_{s}$ did not converge in the first iteration, and there was an obvious peak and trough in the gray error image; in contrast, ${\hat{\phi }}_{s}$ converged in the fourth iteration, and the position deviation was close to zero. However, because of the image noise, there will be many little peaks and troughs, and the fluctuation in the image was caused only by image noise.

**Figure 10:**
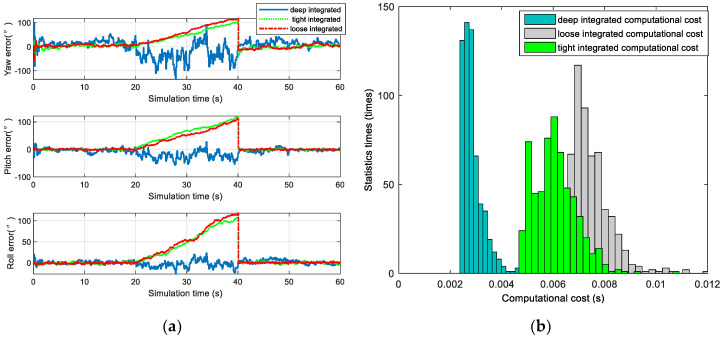
Comparison of different integrated models: (**a**) Accuracy comparison of loosely, tightly, and deeply integrated navigation modes (the optical axis attitude is the Yaw and the non-optical axis attitude is Pitch and Roll); and (**b**) computational cost comparison of loosely, tightly, and deeply integrated navigation modes.

The accuracies and calculation times of loosely, tightly, and deeply integrated modes are presented in Figure 10.

The simulation results show that the attitude accuracies of the loosely, tightly, and deeply integrated navigation modes were of the same magnitude when the number of navigation stars was sufficient, the non-optical axis attitude accuracy was 10” (3 σ), and the optical axis attitude accuracy was 50” (3 σ). The loosely and tightly integrated navigation modes could not identify the navigation stars when the number of navigation stars was insufficient, such that the accuracy became divergent. In contrast, the deeply integrated navigation mode could still be used for the navigation solution when the non-optical axis attitude accuracy was about 50” (3 σ) and the optical axis attitude accuracy was about 100” (3 σ).

Each star occupies about 9 pixels in the star image, and the deeply integrated mode needs 4 iterations. On average, each star needs 36 iterations; the tightly and loosely integrated mode require star identification, there are 196 navigation stars in a sub catalogue on average, and the argument search space is 196×196. The searches number of per star is 196×196/2=19208. Obviously, the computation time of star identification is much larger than the time to update the measurements in deeply integrated navigation filter. The simulation results in Figure 10b show that the computational cost of the deeply integrated navigation mode was 50% lower than that of the tightly integrated navigation mode and 60% lower than that of the loosely integrated navigation mode.

#### 4.1.3. Comparative Simulation of Single- and Double-Star Sensor Configurations

**Figure 11:**
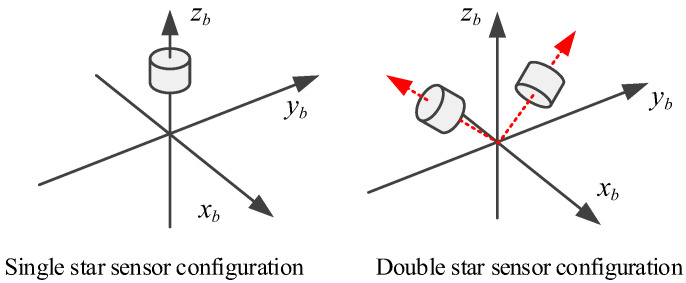
Configuration schemes of star sensors.

The attitude accuracy of the star sensor on the optical axis was lower than the remaining two axes, so celestial navigation systems of near space vehicles should be configured with double star sensors. The star sensor configuration schemes are shown in Figure 11.

**Figure 12:**
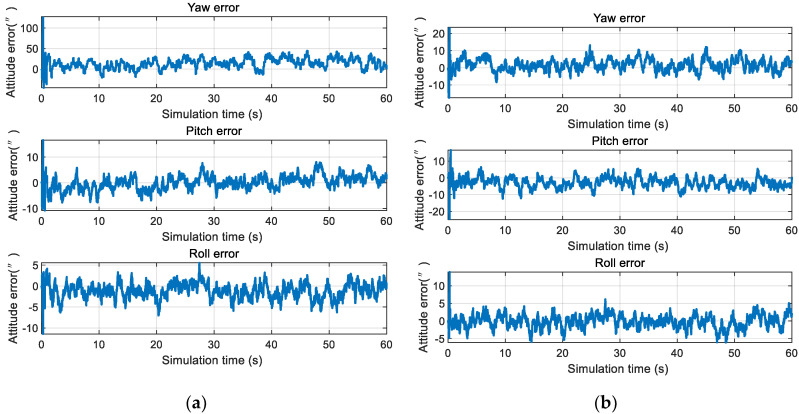
Simulation results of INS/CNS deeply integrated navigation: (**a**) Single-star sensor configuration; and (**b**) double-star sensor configuration.

An accuracy comparison of the single- and double-star sensor configurations is shown in Figure 12.

As shown in Figure 12, the attitude accuracy of the yaw under the single-star sensor configuration was 50” (3 σ), which was much lower than the other two axes. The attitude accuracy of the three axes was kept within 10” (3 σ) under the double-star sensor configuration, which effectively reduced the yaw angle error. For the near space vehicle, the accuracy of the yaw angle had a great influence on the position and, so, it is recommended that the double-star sensor configuration is adopted in celestial navigation systems.

### 4.2. Simulation Verification of the Second-Order State Augmented H-Infinity Filter

#### 4.2.1. Comparison of the Second-Order State Augmented H-Infinity Filter and Standard H-Infinity Filter

**Figure 13:**
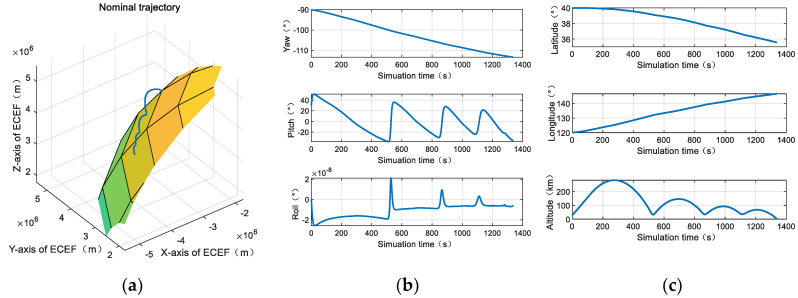
The trajectory of near space vehicles: (**a**) Nominal trajectory; (**b**) the vehicle attitude profile; and (**c**) the vehicle position profile.

The vehicle attitude profile, star sensor and gyro specification, initial attitude estimation error are shown in Figure 13 and Table 1.

**Figure 14:**
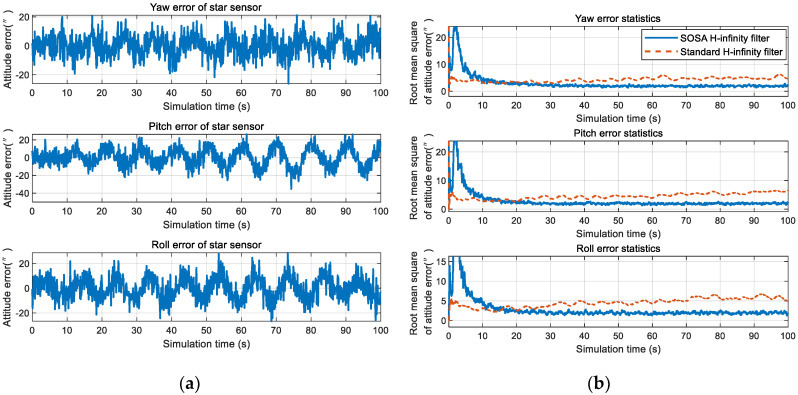
Comparison of the filtering accuracy: (**a**) Measurement error of the star sensor; and (**b**) accuracy comparison of the second-order state augmented H-infinity filter and standard H-infinity filter.

The second-order state augmented H-infinity filter and standard H-infinity filter were compared in a near space environment. As can be seen from Figure 13, the angular velocity of the vehicle is largest in 500 s–600 s, and the 100 s data is used for simulated in Figure 14.

At the beginning of filtering, the SOSA filter exhibit a large error at around 3 s, this is because the initial value of $V_{c,k-1}$ and $V_{c,k-2}$ was unknown, which has to be set to zero at the beginning. Due to $V_{c,k}=\alpha_{1}V_{c,k-1}+\alpha_{2}V_{c,k-2}+\xi_{k}$, if $V_{c,k-1}=0_{3\times 1}$, $V_{c,k-2}=0_{3\times 1}$, $V_{c,k}$ will also close to zero. At this time, the state augmented model cannot estimate the colored noise effectively. In addition, because the estimated value of $V_{c,k}$ is close to zero, the filter will assume the colored noise to be very small (although it is actually not); then, the filter will allocate the error caused by $V_{c,k}$ to other navigation parameters, so the filter will exhibit a large error at around 3 s. However, as the filtering goes on, the colored noise $V_{c,k}$ will be estimated gradually, the state augmentation model will gradually play a role, and finally the navigation accuracy will be improved. Therefore, the filtering results should be used at least 10 s after the beginning of filtering. It can be seen from the simulation that the standard H-infinity filter did not filter out the colored noise; however, the second-order state augmented H-infinity filter proposed in this paper was able to effectively improve the colored noise filtering accuracy. Compared with the standard H-infinity filter, it is more suitable for the near space environment.

#### 4.2.2. The Influence of Colored Noise Model Error on the Filtering Effect

**Figure 15:**
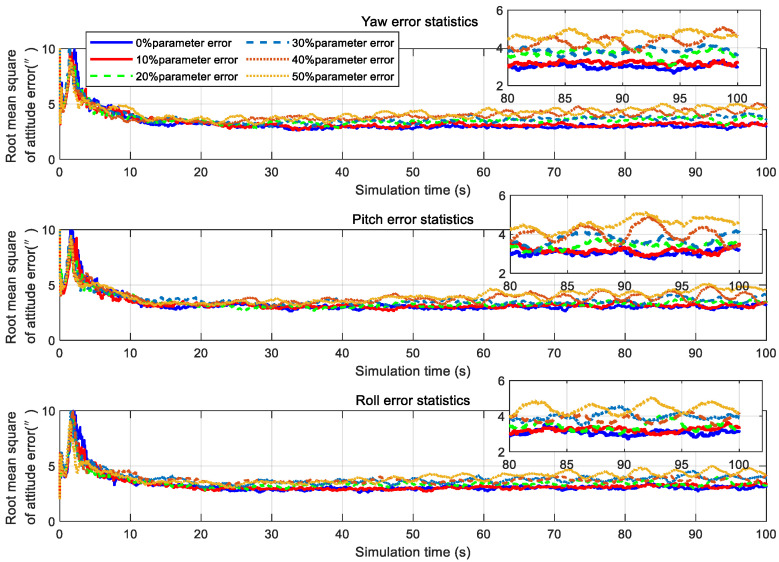
Statistical results of filtering accuracy under various working conditions.

The core of the second-order state augmented H-infinity filter is the colored noise model, where the core parameter of the colored noise model is the digital angular frequency, ω, of the aero-optical effects, whose influences are further illustrated in Figure 15. The attitude root mean square of the second-order state augmented H-infinity filter was about 3” when there was no parameter error. There was little change in the attitude accuracy when the parameter error was 10%, indicating that a 10% parameter error did not have a significant impact on the filtering accuracy. When the parameter error increased to 30%, the attitude root mean square decreased to about 4”. Even when the parameter error increased to 50%, the attitude root mean square did not exceed 5” and, so, the parameter error of the digital angular frequency ω does not significantly affect the second-order state augmented H-infinity filter.

## 5. Conclusions

In this paper, an INS/CNS deeply integrated navigation method was presented for near space vehicles. This method does not need star identification and can significantly reduce the required computational cost. Meanwhile, the proposed second-order state augmented H-infinity filter can weaken the influence of aero-optical effects on the measurement noise and effectively improve the filtering accuracy in near space environments. The simulation results show that the attitude accuracy of the INS/CNS deeply integrated navigation method is kept within 10” (3 σ), while the computational cost can be reduced by 50%. The INS/CNS deeply integrated navigation method therefore can assist in improving the navigation accuracy of near space vehicles and reducing the computational cost of the associated navigation systems, providing a theoretical reference for the design of the near space vehicle navigation systems in the future.

## Figures and Tables

**Table 1:** The star sensor and gyro specification.

Parameter	Value
Field of star sensor	10°
Resolution of star sensor	256 * 256
Measurement noise of star sensor	20”(3 σ)
Gyro bias	0.1 °/h
Gyro noise	0.5 °/h (3 σ)
Initial attitude estimation error	20”(3 σ) ^1^

^1^ After the initial star identification.
